# Supplementary material for: Combined Transcriptomic and Metabolomic Analysis Reveals the Role of Phenylpropanoid Biosynthesis Pathway in the Salt Tolerance Process of Sophora alopecuroides
Source: Int J Mol Sci. 2021 Feb 27;22(5):2399. doi: 10.3390/ijms22052399 (PMC7957753; doi:10.3390/ijms22052399)
Supplement: Supplementary file 1 [file ijms-22-02399-s001.pdf]

Table S1 Summary of Sample sequencing data quality

sample	raw_reads	clean_reads	clean_bases	error_rate	Q20	Q30	GC_pct
CK_1	23313954	22226559	6.67G	0.02	98.21	94.42	43.9
CK_2	23220577	22178023	6.65G	0.02	98.22	94.38	43.24
CK_3	22869484	22042152	6.61G	0.03	98.01	93.83	43.42
T4_1	22355069	21527193	6.46G	0.02	98.18	94.29	43.6
T4_2	20404002	19610694	5.88G	0.03	98.09	94.03	43.46
T4_3	22514859	21636674	6.49G	0.03	98.01	93.88	43.5
T24_1	23155574	22404348	6.72G	0.03	98.09	94.07	43.76
T24_2	23675738	22901075	6.87G	0.03	97.8	93.37	43.58
T24_3	22927443	21985614	6.6G	0.03	98.05	94.04	43.77
T48_1	23198775	22409737	6.72G	0.03	97.99	93.89	43.52
T48_2	22845598	22107440	6.63G	0.02	98.17	94.26	43.49
T48_3	22698775	21813870	6.54G	0.03	97.96	93.85	43.6
T72_1	22971748	22716883	6.82G	0.01	97.03	93.06	43.18
T72_2	23046137	22645899	6.79G	0.01	97.7	94.19	43.46
T72_3	23318379	22999675	6.9G	0.01	97.75	94.27	43.43

Table S2 Statistical results of differential genes

compare	all	up	down	threshold
T4vsCK	2953	1764	1189	DESeq2 padj<0.05 |log2FoldChange|>1
T24vsCK	2218	1133	1085	DESeq2 padj<0.05 |log2FoldChange|>1
T48vsCK	834	378	456	DESeq2 padj<0.05 |log2FoldChange|>1
T72vsCK	21639	16814	4825	DESeq2 padj<0.05 |log2FoldChange|>1
T24vsT4	609	316	293	DESeq2 padj<0.05 |log2FoldChange|>1
T48vsT24	194	24	170	DESeq2 padj<0.05 |log2FoldChange|>1
T72vsT48	18760	15594	3166	DESeq2 padj<0.05 |log2FoldChange|>1

Table S3 Statistical analysis of KEGG pathway enriched by differentially expressed genes

group	ID	pathway_term	rich_factor	qvalue	gene_number
T4_vs_CK	ko04075	Plant hormone signal transduction	0.122137405	7.08E-20	48
	ko00940	Phenylpropanoid biosynthesis	0.099337748	2.50E-10	30
	ko00910	Nitrogen metabolism	0.114864865	1.02E-06	17
	ko00906	Carotenoid biosynthesis	0.192307692	8.36E-06	10
	ko00500	Starch and sucrose metabolism	0.054421769	1.71E-05	32
	ko00052	Galactose metabolism	0.076923077	2.58E-05	19
	ko00945	Stilbenoid, diarylheptanoid and gingerol biosynthesis	0.25	6.37E-05	7
	ko00904	Diterpenoid biosynthesis	0.260869565	0.0002222	6
	ko00250	Alanine, aspartate and glutamate metabolism	0.051515152	0.005626	17
	ko00908	Zeatin biosynthesis	0.166666667	0.005626	5
	ko00941	Flavonoid biosynthesis	0.117647059	0.0077545	6

	ko00040	Pentose and glucuronate interconversions	0.052434457	0.0118706	14
	ko04626	Plant-pathogen interaction	0.040669856	0.0455227	17
T24_vs_CK	ko04075	Plant hormone signal transduction	0.1043257	4.20E-20	41
	ko00940	Phenylpropanoid biosynthesis	0.099337748	2.96E-14	30
	ko04626	Plant-pathogen interaction	0.055023923	2.30E-06	23
	ko00040	Pentose and glucuronate interconversions	0.063670412	1.32E-05	17
	ko00500	Starch and sucrose metabolism	0.040816327	0.0001134	24
	ko00910	Nitrogen metabolism	0.074324324	0.0002245	11
	ko00904	Diterpenoid biosynthesis	0.173913043	0.0063015	4
	ko00908	Zeatin biosynthesis	0.133333333	0.0134926	4
	ko00360	Phenylalanine metabolism	0.049689441	0.0257011	8
T48_vs_CK	ko00040	Pentose and glucuronate interconversions	0.071161049	3.70E-13	19
	ko04075	Plant hormone signal transduction	0.040712468	6.95E-08	16
	ko00940	Phenylpropanoid biosynthesis	0.036423841	3.64E-05	11
	ko00500	Starch and sucrose metabolism	0.025510204	3.64E-05	15
	ko00904	Diterpenoid biosynthesis	0.130434783	0.0047809	3
	ko04626	Plant-pathogen interaction	0.0215311	0.0082429	9
	ko00910	Nitrogen metabolism	0.033783784	0.0154699	5
	ko00906	Carotenoid biosynthesis	0.057692308	0.0266419	3
T72_vs_CK	ko03010	Ribosome	0.317140238	7.99E-20	1038
	ko00940	Phenylpropanoid biosynthesis	0.397350993	3.00E-05	120
	ko00941	Flavonoid biosynthesis	0.549019608	0.0097497	28
	ko00500	Starch and sucrose metabolism	0.307823129	0.0097497	181
	ko04626	Plant-pathogen interaction	0.311004785	0.0366275	130

Table S4 Metabolite difference screening results

Compared Samples	Num. of Total Ident.	Num. of Total Sig.	Num. of Sig.Up	Num. of Sig.down
T24.vs.CK_pos	666	127	110	17
T48.vs.CK_pos	666	155	113	42
T72.vs.CK_pos	666	200	154	46
T48.vs.T24_pos	666	44	20	24
T72.vs.T24_pos	666	98	58	40
T72.vs.T48_pos	666	20	10	10
T24.vs.CK_neg	432	61	48	13
T48.vs.CK_neg	432	82	52	30
T72.vs.CK_neg	432	109	59	50
T48.vs.T24_neg	432	19	11	8
T72.vs.T24_neg	432	47	18	29
T72.vs.T48_neg	432	22	6	16

VIP > 1.0，FC > 1.5, FC < 0.667, P value< 0.05

Table S5 QRT-PCR primers

Gene	Forward primer (5' to 3')	Reverse primer (5' to 3')
Actin	GTCCTTTCAGGAGGTACAACC	CCACATCTGCTGGAAGGTGC
SaPAL2	ATGCTACCTTTGGCTTGA	CCACCATACGCTTTACCT
SaC4H3	ATAAGAATGAGTCGCCAAAT	GGAAGGGAAGAGGAACAC
SaCOMT1	GTCGGATTGGCTCACATT	GATACTCGGCTTCTGGTT
Sa4CL3	CAACCGAGTATTCTTCAT	GCTGCTACTCTTGCTCTT
SaCCR	TGTGGCAGTTGATGAGTC	TACATCCCATGCTGTTTT
SaCADH	TTGGAGGGAGAAGTGCTA	CTTGAAAGACAACGGATT
SaPOD5	TTGGTGGCATTATCTGGT	AGGCGTCTATGTTGGTCT
SaUGT	AATGGTCTTGTGCTGCTT	TCCAAGTTCCACCGTCCC
SaHCT2	GGCTGGTGGTGGCTTGTA	TGGCTCGTGGCCTAGATG
SaCYM	GACTTTAGGCTACTGCCATTC	CTCCTCAGGCTTCATTCC
SaFHD	CACCCTGGGAACACTAAG	GTTGAATCCGACCTACCT
SaANS	TAAGCGTGACTTGTCCAT	TTTCTCCAGCCTCCCATC
SaCHS5	GGTTATGTTTAGTGGGTTG	CCAGATAGTGATGGAGCA
SaCHIS2	CAGTTGGGAGTTATGGTG	AGGTGATTGCCTGTAGAA
SaANR2	TCTAGGTGATGTGGGACT	ACTTTGTTGGAAATACGC
SaFLS	AGAAGGATAAGGGCAGAC	CTTGGGAGGTGAAGAAAT

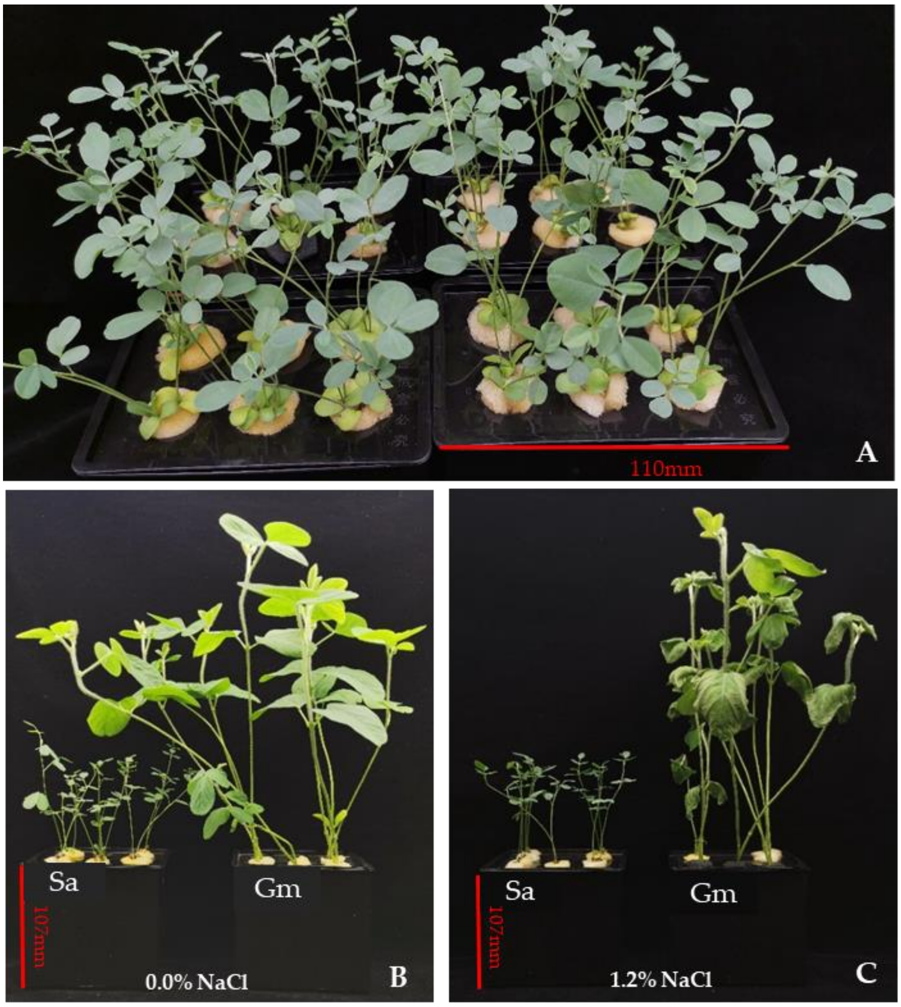

Figure S1 *Sophora alopecuroides* (A) Four-week-old *S. alopecuroides* seedlings; (B) Four-week-old *S. alopecuroides* (Sa) and *Glycine max* (Gm, Williams 82) seedlings with 1/8 Hoagland's nutrient solution; (C) Four-week-old *S. alopecuroides* (Sa) and *Glycine max* (Gm, Williams 82) seedlings with 1.2% NaCl.

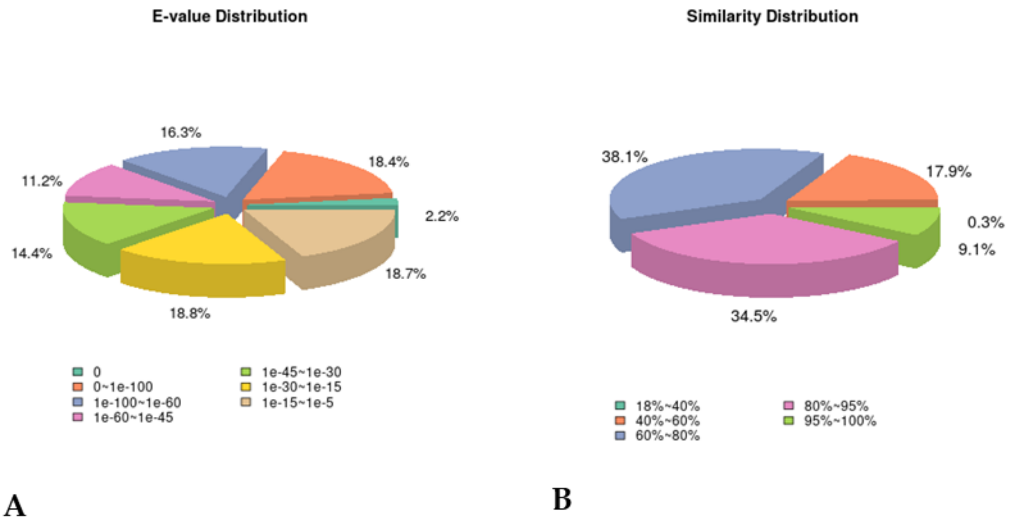

Figure S2. Overview of a time course of *S. alopecuroides* transcriptome responses to salt stress. (A) PCA plots of transcripts identified by RNA-seq of salt-stressed *S. alopecuroides* roots at 0, 4, 24, 48, and 72

hours after stress. (B) Species classification statistics map of the transcriptome NR library comparison of *S. alopecuroides*. (C) KOG annotated classification chart. The X axis is the names of the 26 KOG groups, and the Y axis is the proportion of the number of genes annotated to the group to the total number of genes annotated.

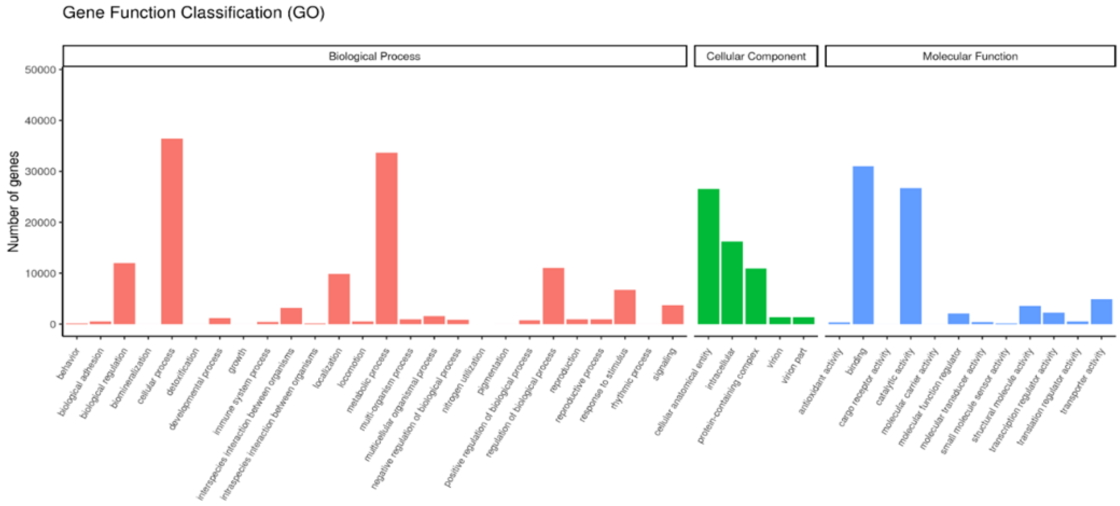

Figure S3. GO annotation classification chart. The X-axis is GO Term, and the Y-axis is the number of genes annotated to the Term.

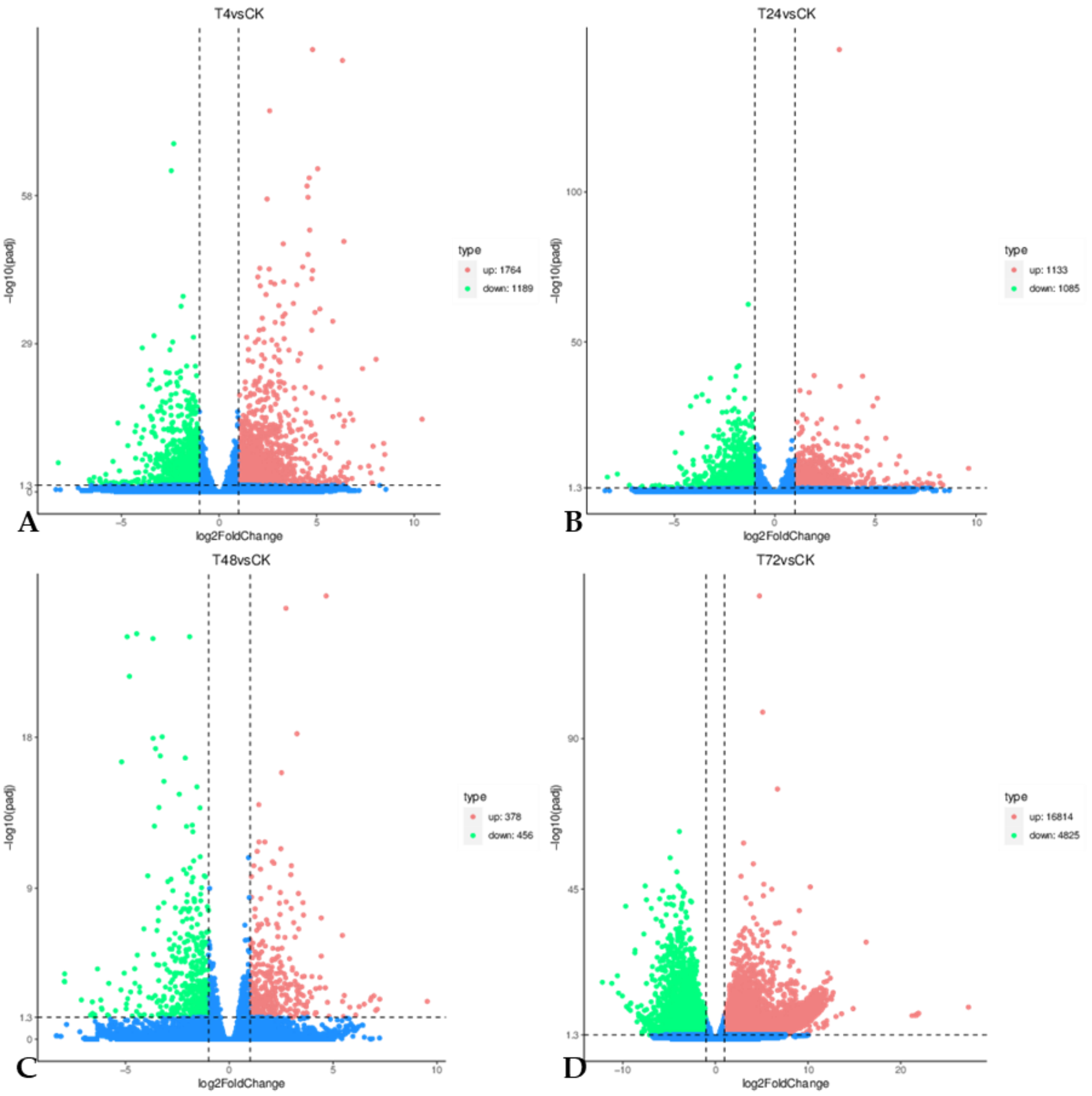

Figure S4. Differential gene volcano map. The abscissa is the log2FoldChange value, the ordinate is -log10pvalue, and the blue dotted line represents the threshold line of the differential gene screening criteria.

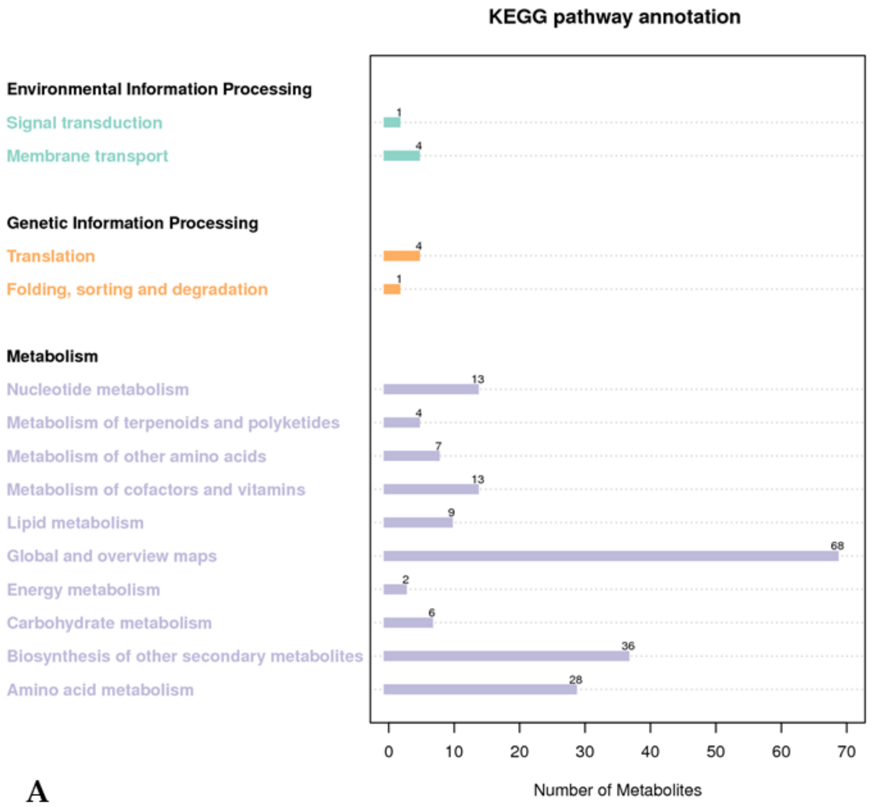

A

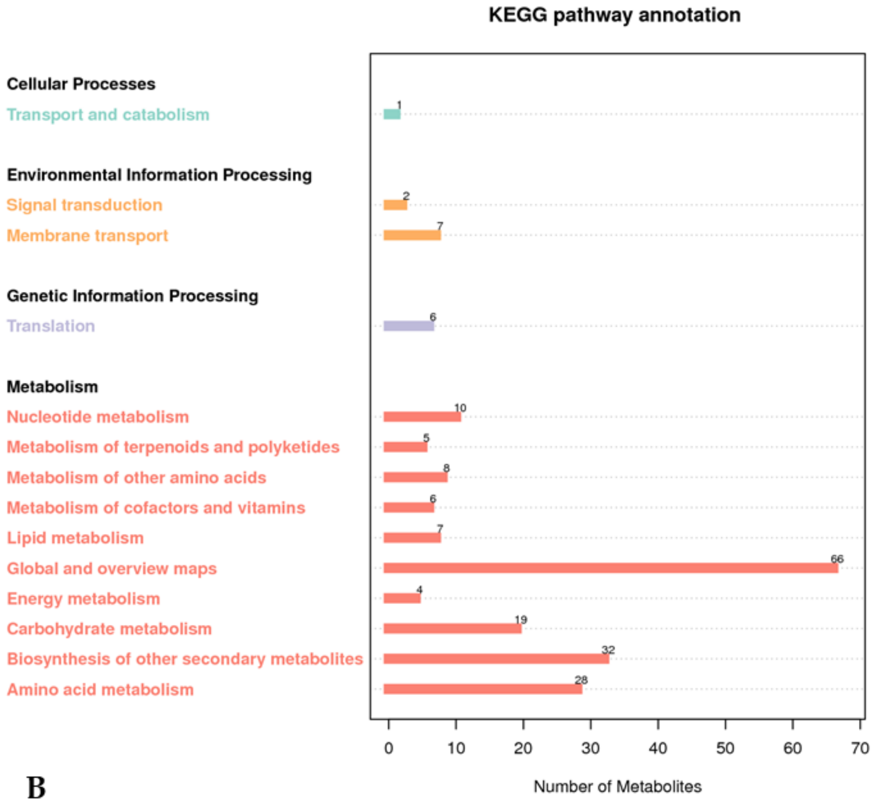

B

Figure S5. KEGG pathway notes (A, positive ion mode; B, negative ion mode). The abscissa represents the number of metabolites, and the ordinate represents the annotated KEGG pathway; it shows the number of metabolites annotated in each second-level classification of Pathway.
